# Endothelial TrkA coordinates vascularization and innervation in thermogenic adipose tissue and can be targeted to control metabolism

**DOI:** 10.1016/j.molmet.2022.101544

**Published:** 2022-07-11

**Authors:** Alexes C. Daquinag, Zhanguo Gao, Yongmei Yu, Mikhail G. Kolonin

**Affiliations:** The Brown Foundation Institute of Molecular Medicine, University of Texas Health Science Center, Houston, TX 77030, USA

**Keywords:** Brown adipose thermogenesis, Neurovascular, Lipolysis, Peptide, TrkA, NGF

## Abstract

**Objective:**

Brown adipogenesis and thermogenesis in brown and beige adipose tissue (AT) involve vascular remodeling and sympathetic neuronal guidance**.** Here, we investigated the molecular mechanism coordinating these processes.

**Methods:**

We used mouse models to identify the molecular target of a peptide CPATAERPC homing to the endothelium of brown and beige AT.

**Results:**

We demonstrate that CPATAERPC mimics nerve growth factor (NGF) and identify a low molecular weight isoform of NGF receptor, *TrkA,* as the CPATAERPC cell surface target. We show that the expression of truncated endothelial *TrkA* is selective for brown and subcutaneous AT. Analysis of mice with endothelium-specific *TrkA* knockout revealed the role of TrkA in neuro-vascular coordination supporting the thermogenic function of brown adipocytes. A hunter-killer peptide D-BAT, composed of CPATAERPC and a pro-apoptotic domain, induced cell death in the endothelium and adipocytes. This resulted in thermogenesis impairment, and predisposed mice to obesity and glucose intolerance. We also tested if this treatment can inhibit the tumor recruitment of lipids mobilized from adipocytes from adjacent AT. Indeed, in a mouse model of breast cancer D-BAT suppressed tumor-associated AT lipolysis, which resulted in reduced fatty acid utilization by cancer cells.

**Conclusion:**

Our study demonstrates that *TrkA* signaling in the endothelium supports neuro-vascular coordination enabling beige adipogenesis.

## Highlights

• Endothelial TrkA isoform expression is selective for brown and beige adipose tissue.
• Endothelial TrkA isoform TrkA coordinates neuro-vasculature promoting thermogenesis.
• Peptide D-BAT mimicking NGF targets Endothelial TrkA in brown/beige adipose vasculature.
• D-BAT causes brown/beige adipose tissue reorganization and suppresses its function.
• Cancer-induced adipose tissue lipolysis and beiging is suppressed by D-BAT.

## 1 Introduction

An important function of adipose tissue (AT) is the maintenance of energy balance that is fundamental for healthy metabolism [1]. The ability of adipocytes in white AT (WAT) to store and metabolize lipids predetermines susceptibility to type-2 diabetes and other components of the metabolic syndrome and its complications [2]. Visceral AT (VAT) mainly stores lipids, and its excessive expansion, inflammation, and dysfunction in obesity is linked with metabolic disease [3]. In contrast, subcutaneous AT (SAT) can protect from metabolic disease [1,4]. In response to sympathetic nervous system (SNS) stimuli such as cold exposure, SAT acquires mitochondria-rich adipocytes specialized to activate lipolysis and burn lipids through adaptive thermogenesis [5]. The organ specialized in executing this function is brown AT (BAT), which has fixed anatomic locations [[6], [7], [8]]. The “inducible/recruitable” brown-like (beige aka brite) adipocytes, arising in WAT in response to catecholamines activating β-adrenergic receptors, are functionally similar to brown adipocytes in the canonical (constitutive) BAT [9,10]. The function of brown and beige adipocytes is muscle-independent thermogenic energy dissipation, which relies on the function of uncoupling protein 1 (UCP1), which leaks protons to uncouple substrate oxidation from ATP synthesis, resulting in heat dissipation [1,11]. Both BAT and beige adipocytes activate energy expenditure and can counteract metabolic consequences of obesity in mice [12,13]. In humans, brown adipocytes are present in various AT depots perinatally, during puberty and pregnancy [5], and are common in the subclavicular and supraclavicular area in adulthood [14].

While brown adipocytes may be metabolically beneficial in obesity [5,15], their hyperactivation can have adverse effects. Excessive energy expenditure due to catecholamine deregulation underlies hypermetabolic conditions that first lead to excessive AT lipolysis and subsequently to cachexia and life-threatening wasting of lean body mass [[16], [17], [18], [19]]. In severe burn injury patients, AT browning is activated in response to breached thermoregulation and inflammation, and results in diabetes [20,21]. In cancer patients, growth of some types of tumors induces adrenergic stimuli that cause local lipolysis and AT browning in mice [16,[22], [23], [24]]. Increased brown AT activity has been observed in human pediatric cancers [[25], [26], [27]], and there are reports indicating AT browning in adult human cancer patients [28]. Approaches to selectively inactivate AT browning could help to control these pathogenic conditions.

Adipocytes, as well as other cell types composing AT stroma and vasculature, execute distinct functions controlling tissue metabolism [1]. Vascular angiogenesis has been shown to accompany AT conversion from white to beige [29]. Changes in endothelial cells (EC) underlying AT beiging, or its conversion back to WAT, affect sympathetic innervation through mechanisms that are poorly understood. We have previously established an approach to interrogate functions of organ-specific vascular beds based on peptides binding selectively expressed endothelial cell surface molecules [[30], [31], [32]]. We have hypothesized that targeting AT vasculature in BAT and beige AT could be used to modulate adipocyte activity. In a screen for peptides that home to BAT in mice, we previously isolated a cyclic peptide termed PEP3 (amino acid sequence CPATAERPC) that homes to the endothelium of BAT and beige SAT upon systemic administration [33].

Here, we identify the cell surface molecule bound by PEP3 as nerve growth factor receptor *NTRK1*, also known as tropomyosin receptor kinase A (*TrkA*). We validate *TrkA* as the *PEP3* target by demonstrating that PEP3 does not home to BAT and beige SAT in mice that lack TrkA in the endothelium. Our results indicate that TrkA signaling in AT endothelium is dispensable for vascularization during development but supports the alignment of nerves and vessels and, hence, the thermogenic function of adipocytes in BAT and beige AT. To investigate whether PEP3 could be used for experimental inactivation of BAT and beige AT, we designed a hunter-killer peptide D-BAT, composed of PEP3 and a pro-apoptotic domain. Using mouse models, we demonstrate that apoptosis in brown and beige fat vasculature caused by D-BAT results in adipocyte death and partial resorption of these tissues, while white adipocytes are spared. We show that D-BAT treatment decreases cold tolerance, metabolic rate, lipid utilization for energy, and glucose clearance. As a result, mice treated with D-BAT are more prone to development of obesity induced by high-fat diet (HFD), a phenotype expected of suppression of BAT and beige AT function. Finally, we show that AT browning and lipolysis induced by tumor growth can be suppressed by D-BAT treatment.

## 2 Materials and METHODS

### 2.1 Animal housing and analysis

Studies were approved by and performed according to the guidelines of the Institutional Animal Care and Use Committee of UTHealth. C57BL/6 mice were purchased from Jackson. TIE2e-Cre and TrkA^fl/fl^ strains in C57BL/6 background were crossed and genotyped as described [34,35]. Mice were housed in the animal facility with a 12-h light/dark cycle and room temperature and had free access to water and diet. Body weight, body composition, cold tolerance test, glucose tolerance test and energy expenditure were measured before and after the three months of HFD (Research Diets D12331, 58 kcal% fat) feeding. Physiological tests were performed as we previously described [24,31,[36], [37], [38], [39], [40]]. Body weight was measured weekly. Body composition (fat and lean mass) was measured using EchoMRI-100 (Echo Medical Systems). Indirect calorimetry studies were performed with OXYMAX (Columbus Instruments) Comprehensive Lab Animal Monitoring System (CLAMS). Food intake was measured over a period of three days. Cold tolerance was measured and tissues were recovered 3 days after the last peptide injection. For cold tolerance test, mice were placed into environmental chamber IS33SD (Powers Scientific) set to 4 °C. Core body temperature was determined using a MicroTherma 2 K High Precision Type K Thermocouple Meter (THS-221-092, ThermoWorks)/RET-3 rectal probe (Braintree Scientific). For glucose tolerance test, glucose (1 g/kg body weight) was injected i.p. Into overnight-fasted mice. For insulin tolerance test, insulin (0.6 U/kg body weight) was injected i.p. Into mice fasted 4 h. Blood glucose concentration was measured with a glucometer (One Touch Ultra).

### 2.2 Tissue analysis

**Figure 1:**
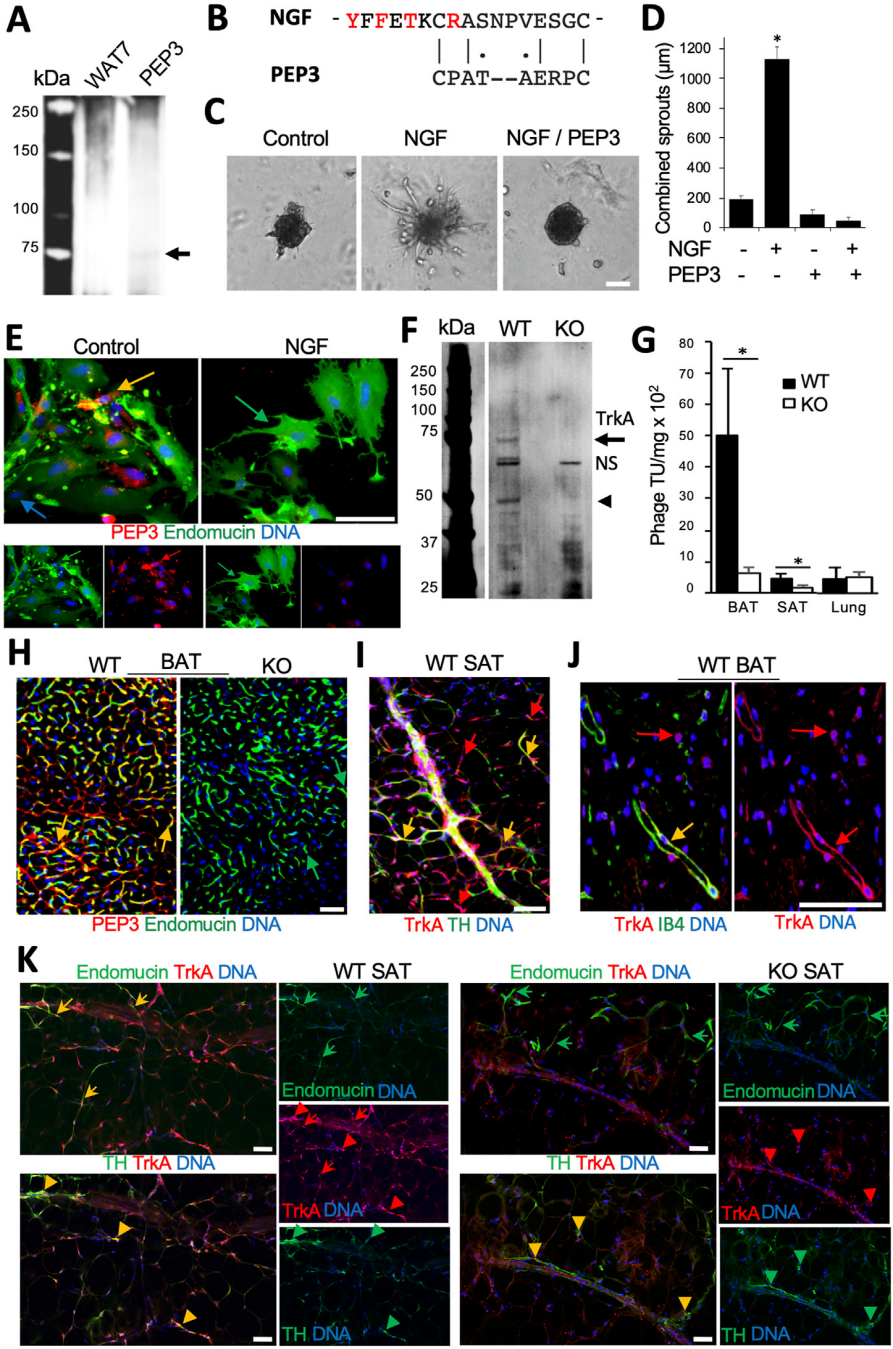
Endothelial TrkA isoform is the target of PEP3 in AT. (**A**) PEP3 receptor isolation from mouse BAT membrane protein extract. Upon elution with PEP3 or negative control peptide (WAT7), the specific band in silver-stained SDS-PAGE gel (arrow) was identified as TrkA. (**B**) Alignment of mouse NGF amino acid stretch 173–189 and PEP3. Red: residues in contact with TrkA. Lines: identity; dots: amino acids of similar class. (**C**) HUVEC sprouting promoted by NGF (50 nM) and blocked by 50 nM of PEP3. (**D**) Data from **C** quantified; Error bars: SEM, ∗P < 0.01 (Student's t-test); N = 5 spheroids. (**E**) PEP3 (biotinylated) binding to EC (endomucin^+^ IF) in SVF from SAT is blocked by equimolar concentration (1 ug/ml) of NGF. Yellow arrow: PEP3-bound EC. Blue arrow: non-endothelial cell (not PEP3-bound. (**F**) PEP3 receptor isolation performed from extracts of WT vs TrkA-EC-KO (KO) mice. Immunoblotting with anti-TrkA antibodies reveals the 70 kDa (arrow) and a smaller (arrowhead) isoforms missing in KO. NS: non-specific bands. (**G**) After 2 × 10^9^ transforming units (TU) of phage displaying PEP3 injected into mice for 1 h, PEP3-phage accumulates more in BAT and SAT of WT mice compared to KO littermates; there is no difference for control organ (lung). Error bars: SD ∗P < 0.05 (Student's t-test); N = 3 phage TU recovery quantifications. (**H**) Paraffin sections of BAT from mice injected with biotinylated PEP3 subjected to strepatavidin-Cy3 and endomucin IF. Note vascular homing of PEP3 in WT but not in KO mice. (**I**) Whole mount of WT SAT demonstrating TrkA IF co-localized (yellow) and not co-localized (red) with nerves (TH+, green). (**J**) Paraffin section of WT BAT demonstrating TrkA IF co-localized (yellow) and not co-localized (red) with blood vessels (IB4^+^, green). (**K**) Serial paraffin sections of SAT subjected to IF with antibodies against TrkA and endomucin (arrows) or TrkA and TH (arrowheads). Note that in WT mice (left) TrkA co-localizes with EC and TH + neurons, while in KO mice (right) TrkA co-localizes only with neurons. DNA is blue; Scale bar: 50 μm.

For tissue analyses, organs were recovered after heart perfusion with 10 ml PBS. Inguinal AT (SAT), gonadal AT (VAT) and interscapular BAT were either fixed in buffered 4% paraformaldehyde and used for whole mounts or 10% formalin-fixed and used for paraffin-embedding and sectioning as we previously described [24,33,40,41]. Hematoxylin and eosin (H/E) staining was performed by histology core. For immunofluorescence (IF) analysis, upon antigen retrieval and section blocking, primary (4 °C, 12 h) and secondary (RT, 1 h) antibody incubations were done with the following antibodies diluted in PBS with 0.05% Tween 20: anti-TrkA: (Bioss, bs-10210 R; 1:100 and BicellScientific 06021; 1:100); anti-endomucin (R&D Systems, AF4666; 1:100); anti-TH (Pel-Freez, P40101; 1:100); anti-UCP1 (Sigma, U6328; 1:400); anti-perilipin1 (Abcam, ab61682; 1:100); anti-cleaved caspase3 (Cell signaling, 9661; 1:100); anti-pHSL (Cell signaling, 4126; 1:300). Donkey Alexa 488-conjugated (1:200) IgG, Cy3-conjugated (1:300) IgG and Streptavidin-Cy3 (1:200) were from Jackson ImmunoResearch. Jackson ImmunoResearch Cy5-conjugated donkey anti-goat IgG (705-175-147) was used at 1:200 and Cy5 fluorescence was pseudo-colored red (In Figure 1K) or green (Supplementary Fig. 3C). Biotinylated isolectin B4 (Vector, B-1205; 1:50) was used with Cy3-or Alexa488-conjugated streptavidin as described. Nuclei were stained with Hoechst 33,258 (Invitrogen, H3569). Images were acquired with a Carl Zeiss upright Apotome Axio Imager Z1/ZEN2 Core Imaging software or with confocal microscopes Nikon AX R/NIS-Elements software or Leica TCS SP5/LAS AF software. Image J analysis software was used to quantify data.

### 2.3 Identification and validation of PEP3 receptor

Peptides were used as a bait for receptor purification as described previously [30,41]. Mouse BAT was disrupted in PBS containing 0.2 mM phenylmethanesulphonylfluoride/Roche protease inhibitor (PI) cocktail with a Dounce homogenizer. Centrifugation (15,000 g for 30 min at 4 °C) was performed to separate membrane pellet, which was solubilized in PBS containing 1 mM CaCl_2_, 1 mM MgCl_2_, 50 mM n-Octyl-beta-d-glucopyranoside and PI cocktail (column buffer). 5 mg of peptide PEP3 [33] and control WAT7 peptide (Daquinag et al., 2011) were coupled (via C-terminus) onto 0.25 ml of EDC Sepharose (Pierce), and the column was equilibrated with column buffer containing 1% Triton X-100. Peptide-coupled resin was incubated with 40 mg of membrane extract and washed with column buffer. Elution (0.5 ml fractions) was performed with 0.1 M glycine (pH 2.8). Mass spectroscopy of the proteins extracted from the PEP3-specific gel band and protein identification was performed by UTHealth Clinical and Translational Proteomics Service Center. For validation, 800 ug of SAT SVF lysate was incubated with 30 ug biotinylated PEP3 peptide bound to streptavidin-coated NHS-Activated Magnetic Beads (Pierce #88826) overnight at 4 °C. The beads were washed with 0.05% Tween-20 TBS, and heated at 95 °C in 30 μl of 1X NuPAGE SDS sample buffer for 8 min. Precipitates and primary tissue extracts were resolved by 12% SDS-PAGE gel and immunoblotted with anti-TrkA antibodies (BicellScientific #06021; 1:1000) and control anti-β-actin (Abcam, ab8224; 1:5000) antibodies. Signal was detected using the Odyssey DLx imaging system (LI-COR) and quantified with Image J analysis software.

### 2.4 Cell culture assays

For adipocyte and stromal/vascular fraction (SVF) cell isolation, SAT pads of 8-week-old mice were excised, minced and digested in 0.5 mg/ml collagenase type I (Worthington Biochemical, LS004196) and 2.5 mg/ml of dispase Roche, 04942078001) solution in a shaking bath for 1 h at 37 °C. The cell suspension was filtered through a 70 μm cell strainer (Thomas Scientific, 1181X53) followed by centrifugation (360×*g*, 5 min, RT). Cells were isolated and cultured in DMEM supplemented with 10% fetal bovine serum and penicillin–streptomycin at 37 °C, 5% CO_2_ as described [32]. Endothelial angiogenic sprouting assay with HUVEC cells (received from ATCC, PCS-100-10), and with mouse AT explants, was performed as previously described [40,42] in EGM-2MV medium (Lonza, CC-4147). NGF was from Sigma (N2513).

### 2.5 Animal tumor models and peptide treatments

**Figure 3:**
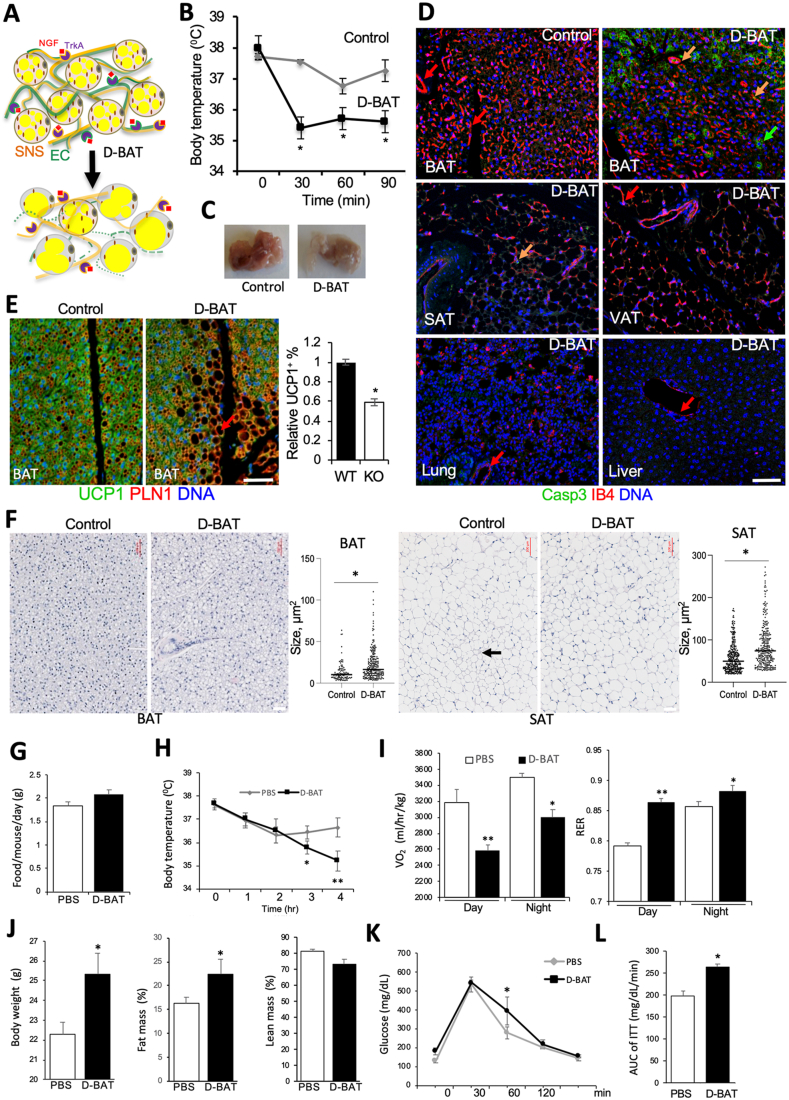
D-BAT induces apoptosis selectively in EC of thermogenic AT. (**A**) A model predicting D-BAT effect in AT. D-BAT depletes EC expressing the PEP3-tsargeted TrkA isoform while sparing SNS. Reduced BAT and SAT vascularization results in AT whitening and reduced thermogenesis. (**B**–**F**) C57BL/6 males were treated with D-BAT or PBS (control) over 2 weeks (N = 5). (**B**) Core body temperature maintenance at 4 °C measured 3 days after the last injection indicating reduced cold tolerance upon D-BAT treatment. Plotted are mean ± SEM; ∗P < 0.05 (Student's t-test). (**C**) Interscapular BAT 3 days after the last injection of D-BAT. (**D**) IF analysis of paraffin tissue sections from D-BAT-treated and control mice with cleaved caspase 3 IF (green) co-localized with IB4 (red). Note apoptosis in both endothelium (yellow arrow) and adipocytes (green arrow) of D-BAT-treated mice. (**E**) BAT IF for UCP1 (green)/perilipin1 (PLN1). Graph indicates decreased frequency of UCP1+ adipocytes, which are partly replaced by white adipocytes in D-BAT-treated mice. (**F**) Representative images of H/E-stained paraffin sections of BAT and SAT from treated and control mice with quantifications to the right. Arrow: beige adipocyte area. In D-F, scale bar: 50 μm. (**G**–**L**) C57BL/6 female mice treated with D-BAT were fed HFD for three months (N = 5). (**G**) Food consumption unchanged by D-BAT. (**H**) Core body temperature maintenance at 4 °C indicating reduced cold tolerance in D-BAT-treated mice. (**I**) Oxygen consumption (VO_2_) during day and night decreased by upon D-BAT treatment. Respiratory exchange ratio (RER) increase indicates that D-BAT treatment leads to lower lipid oxidation. (**J**) Body mass and EchoMRI indicates fat body mass increase in mice treated with D-BAT. (**K**) Fasted glucose tolerance test. (**L**) Insulin tolerance test (ITT) area under the curve (AUC). Plotted are mean ± SEM; ∗*P* < 0.05, ∗∗*P* < 0.01 (Student's t-test).

**Figure 4:**
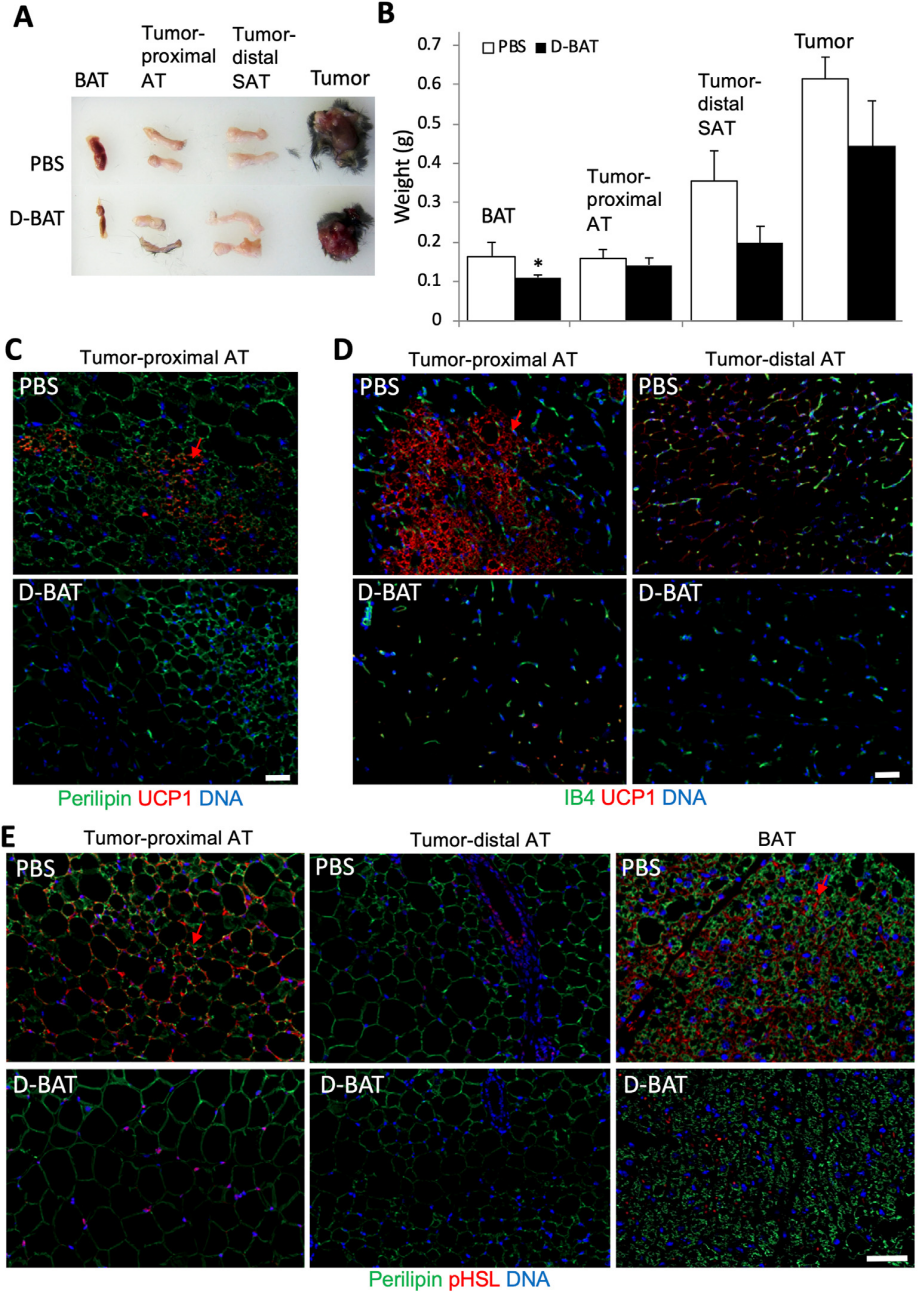
D-BAT attenuates tumor-associated AT browning and lipolysis. C57BL/6 female mice (N = 5), pre-treated with D-BAT or PBS (control) over 3 weeks, were grafted with tumors and then injected with D-BAT or PBS for 2 more weeks. (**A**) BAT, tumor-distal SAT, tumor-proximal VAT and tumors from representative mice. (**B**) Quantification of tissue mass from all mice (N = 5). Plotted are mean ± SEM; ∗*P* < 0.05, (Student's t-test). (**C**) IF with UCP1/red fluorescence secondary antibodies and perilipin1/green fluorescence secondary antibodies. Arrow: beige AT. (**D**) IF analysis with UCP1/red fluorescence secondary antibodies and co-stained with isolectin B4 (green) marking endothelium. Arrow: beige AT. (**E**) IF with phosphorylated hormone-sensitive lipase (pHSL)/red-fluorescent secondary antibodies and perilipin1/green-fluorescent secondary antibodies. Note browning (UCP1 expression) and lipolysis (pHSL expression, arrows) in BAT and tumor-proximal AT, which is reduced by D-BAT treatment but not by PBS. Nuclei are blue. Scale bars: 50 μm.

Peptide _d_CPATAERPC-_d_KLAKLAKKLAKLAK (D-BAT) was designed based on previously described strategy [30,43] with all amino acids as d-enantiomers and with aminohexanoic acid NH(CH_2_)_5_CO as a linker (−). It was synthesized by peptide chemistry, cyclized via cysteines, purified to >95% purity by HPLC and quality-controlled (mass spectroscopy) by Celtek Peptides. Peptide acetate salt powder was dissolved in phosphate-buffered saline (PBS) to 10 mM and aliquots were stored frozen until dilution in PBS, filtration, and use. Biotinylated PEP3 (500 μg) or 2 × 10^9^ transforming units (TU) of phage-PEP3 were injected via tail vein and left to circulate for 1 h. D-BAT was administered by metronomic subcutaneous (sc) injections on the alternating sides of lower back based on optimized protocol [31,32]. For experiment in 2-month-old male mice (Figure 3), seven 0.2 ml D-BAT (1 mM) injections were performed over a period of 2 weeks. To investigate long-term effects of D-BAT (Figure 3), 2-month-old female mice fed chow received ten 0.1 ml injections of D-BAT (0.5 mM) over a period of three weeks. Then the mice were switched to feeding HFD for diet-induced obesity (DIO) induction, and six more 0.1 ml injections of D-BAT (0.5 mM) were given over two subsequent weeks. To investigate the effects of D-BAT on cancer-induced AT browning (Figure 4), 2-month-old female mice fed chow received ten 0.1 ml injections of D-BAT (0.5 mM) over a period of three weeks. Then, 1 × 10^5^ E0771 cells were grafted with a 21-gauge needle into the mammary fat pad as described [32] and six more 0.1 ml injections of D-BAT (0.5 mM) were given over two subsequent weeks. Tumor size was measured with a caliper weekly and tumors were weighed upon resection.

### 2.6 Statistics

Statistical analyses were performed using Microsoft Excel. Experimental groups were compared by using two-tailed unpaired Student's t-test. P values lower than 0.05 were considered significant. All results are presented as mean +/− SEM or SD as indicated.

## 3 Results

### 3.1 TrkA is the endothelial target of BAT-homing peptide PEP3

To purify the PEP3 receptor, we used an established biochemical approach previously used by our group [30,41]. PEP3 and a negative control peptide WAT7 [41], covalently attached to a resin, were incubated with membrane protein extract of mouse BAT. After washing off unbound proteins, elution was performed. Polyacrylamide gel electrophoresis (*PAGE*) of eluted proteins revealed a band of ∼70 kDa specifically observed for PEP3 elution but not for the control elution (Figure 1A). Mass spectrometry on the extract from that band revealed the PEP3-bound protein is the nerve growth factor receptor TrkA [44]. The native ligand of TrkA is the nerve growth factor (NGF), which, along with other neurotrophins, promotes survival and proliferation of peripheral sensory and sympathetic neurons [45]. Amino acid sequence alignment revealed a similarity between PEP3 and amino acid stretch 173–189 of mouse NGF (Figure 1B). This segment contains a β-sheet YFFETKCR of NGF that is reported to mediate TrkA binding [44]. This suggested that PEP3 binds TrkA by mimicking NGF. To confirm this, we used human umbilical vein endothelial cells (HUVEC), known to express TrkA and activated by NGF [46]. PEP3, added at equimolar concentration, blocked NGF-induced HUVEC sprouting (Figure 1C), which was quantified by measuring sprout length (Figure 1D). PEP3 homing to the endothelium [33] suggested that TrkA should be expressed in EC of BAT/beige AT. Indeed, experiments with SVF from mouse SAT revealed that PEP3 binds selectively to EC, identified by endomucin IF (Figure 1E). In a competition experiment reciprocal to Figure 1C, PEP3 binding to adipose EC was blocked by NGF, indicating their binding to the same receptor (Figure 1E).

To further validate TrkA as the PEP3 receptor, we generated mice with an endothelial-specific TrkA knockout. Upon crossing TIE2e-Cre [36,37] and TrkA^fl/fl^ [35] strains, we obtained TIE2e-Cre; TrkA^fl/fl^ (EC KO) and control Cre-negative TrkA^fl/fl^ (WT) littermates. Western blotting confirmed that the low molecular weight TrkA isoform, prominent in BAT and detectable in SAT of WT mice, was undetectable in BAT and SAT of KO mice (Supplementary Fig. 1A). In contrast, intensity of the full-length TrkA in the brain was not changed. The observation that the truncated, but not the full-length, TrkA isoform is excised in the floxed mice reiterates the point that only the endothelial TrkA isoform expression is AT depot-specific (Supplementary Fig. 1A). We also performed the PEP3 receptor pull-down experiment with WT and TrkA EC KO AT extracts for side-by-side comparison. Anti-TrkA immunoblotting confirmed precipitation of the 70 kDa TrkA isoform from WT but not from TrkA EC KO extracts (Figure 1F). Systemic administration of phage displaying PEP3 into mice demonstrated that the loss of TrkA was sufficient to significantly reduce the amount of phage-PEP3 particles accumulating in BAT and SAT, while background phage signal in lungs was undistinguishable (Figure 1G). IF confirmed that phage displaying PEP3 does not home to BAT of TrkA EC KO mice (Supplementary Fig. 1B). Nonspecific phage trapping in liver reticulo-endothelial system, comparable in WT and KO mice, confirmed that the difference was not due to a variation in injection efficacy (Supplementary Fig. 1C). Biotinylated PEP3 [33] was also injected for validation of TrkA as the target receptor. Tissue analysis confirmed PEP3 localization in BAT vasculature of WT but not of TrkA EC KO mice (Figure 1H). Upon cell recovery, PEP3 was detected in EC, but not in stromal cells from BAT of WT mice; in contrast, PEP3 binding was not detectable in EC from BAT of TrkA EC KO mice (Supplementary Fig. 1D). Systemically administered PEP3-phage was not co-localized with a sympathetic neuronal marker, tyrosine hydroxylase (TH), indicating that neuronal TrkA is not the target of PEP3 (Supplementary Fig. 1E). Combined, these data confirm the endothelial TrkA isoform as the PEP3 target in BAT and beige AT.

While TrkA signaling is well characterized in neurons [35], there are no reports on its expression in adipose vasculature. Tissue IF analysis with anti-TrkA antibodies demonstrated that, in addition to TH-positive nerves TrkA is expressed in TH-negative cells in both BAT (Supplementary Fig. 1F) and SAT (Figure 1I). Co-localization with isolectin B4 (IB4), marking the endothelium revealed TrkA expression in blood vessels (Figure 1J). Endomucin co-localization confirmed endothelial TrkA expression in BAT (Supplementary Fig. 1G). IF analysis of SVF cells from mouse SAT revealed TrkA expression in some, but not all, IB4-positive EC (Supplementary Fig. 1H). Consistent with the PEP3 homing to BAT and beige AT, TrkA expression was not detectable in the endothelium of VAT (Supplementary Fig. 1I). Analysis of SAT from TrkA EC KO mice demonstrated that expression of TrkA in TH + neurons was unaffected and was selectively missing in endomucin + EC (Figure 1K). This was confirmed by high-power confocal microscopy (Supplementary Fig. 1J) and quantified (Supplementary Fig. 1K).

### 3.2 Endothelial TrkA regulates neuro-vascular alignment and thermogenesis in AT

**Figure 2:**
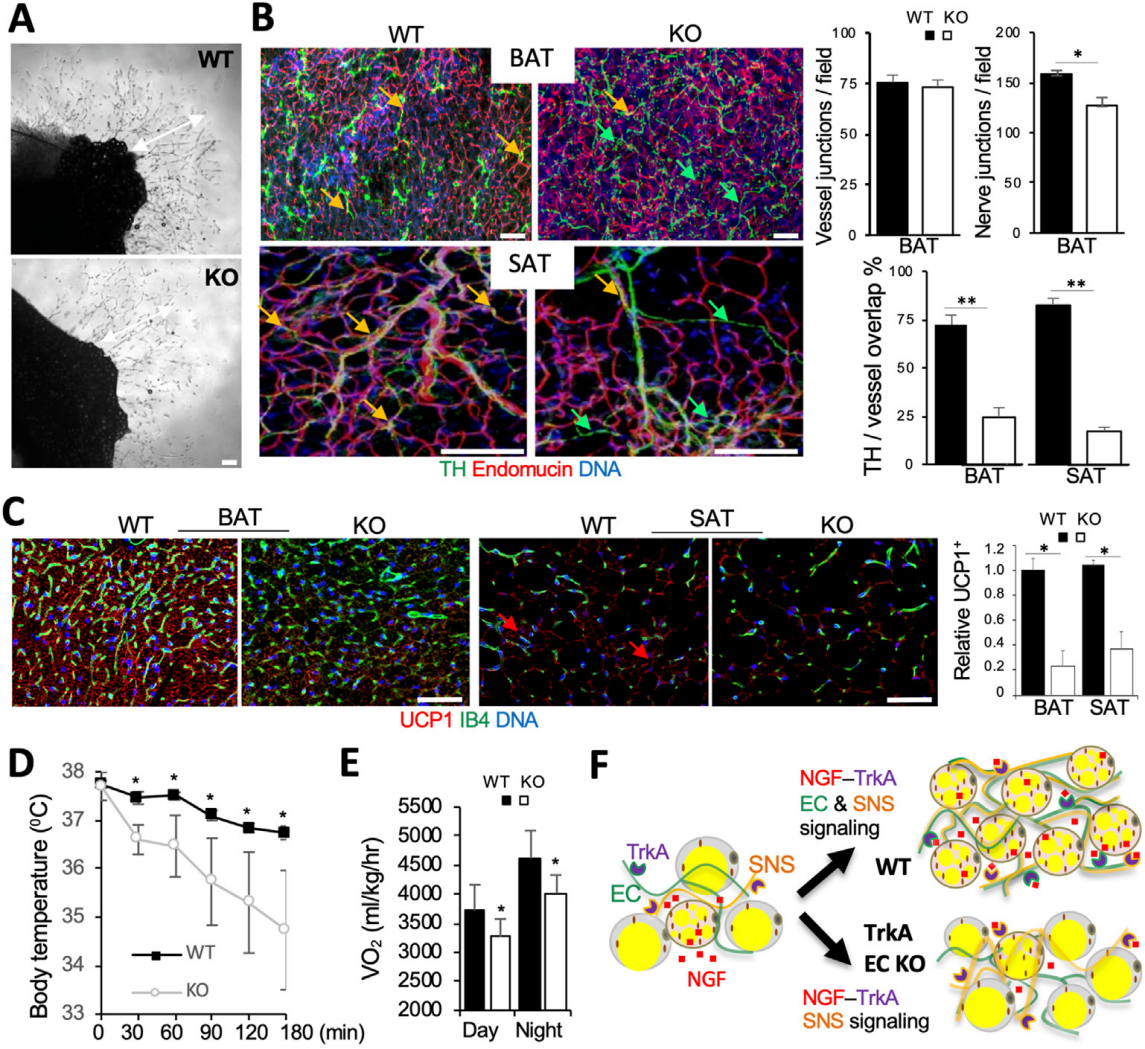
Endothelial TrkA regulates AT neuro-vascular alignment and thermogenesis. (**A**) SAT explants at day 4 of culture show normal angiogenic sprouting (arrows) for TrkA EC KO. (**B**) Whole mounts subjected to IF demonstrating blood vessel (endomucin+) misalignment with nerves (TH+) in BAT and SAT of KO mice. Graphs: data quantification; Error bars: SEM ∗P < 0.05, ∗∗P < 0.01 (Student's t-test); N = 5 view fields. (**C**) Paraffin sections stained with IB4 and subjected to UCP1 IF demonstrating lower UCP1 expression in BAT and lack of beige AT (arrows) in SAT of KO mice. Graphs: relative UCP1 expression quantification; ∗P < 0.01, N = 5 view fields. Error bars: SD ∗P < 0.05 (Student's t-test). In A-C, DNA is blue; Scale bar: 50 μm. (**D**) Cold intolerance of male KO mice vs WT littermates, measured based on core body temperature maintenance at 4 °C. N = 6. Error bars: SD ∗P < 0.05 (Student's t-test). (**E**) Reduced oxygen consumption of EC KO mice during day and night. N = 5. Error bars: SEM ∗P < 0.05 (Student's t-test) calculated based on the analysis of multiple time points (Supplementary Fig. 2D). (**F**) A model of TrkA function in AT endothelium. NGF secreted by brown adipocytes activates TrkA in both SNS nerves and endothelial cells (EC). Endothelial TrkA signaling coordinates angiogenesis with SNS neuronal guidance, resulting in neuro-vascular network conducive of brown adipogenesis. Without endothelial TrkA, vascularization uncoordinated with SNS innervation results in reduced UCP1 expression and reduced thermogenesis.

We then investigated the function of TrkA in adipose endothelium. Comparison of SAT explants from WT and KO littermates did not reveal abnormalities for TrkA-negative EC sprouting in angiogenic medium (Figure 2A). We did not observe a decrease in vascular density in AT of TrkA EC KO mice (Figure 2B). Vascular perfusion was also found to be normal in AT of TrkA EC KO mice (Supplementary Fig. 2A). There was no significant difference in BAT or SAT nerve density between WT and TrkA EC KO littermates (Supplementary Fig. 2B). However, branching of TH + nerves was reduced in BAT of KO mice (Figure 2B). Moreover, while TH + neurons tended to be aligned with vessels in BAT and SAT of WT mice, disconnection of nerves from the vasculature was observed more frequently in KO mice (Figure 2B). Neuro-vascular cooperation is known to be important for the thermogenic function of BAT and SAT [47]. We therefore compared UCP1 expression in AT paraffin sections from TrkA EC KO and WT mice. Concordantly with reduced vascularization, there was a significantly lower UCP1 IF signal in both BAT and SAT of EC KO mice (Figure 2C). Lower UCP1 expression in KO mice was confirmed by Western blotting (Supplementary Fig. 2C). To confirm that UCP1 underexpression results in reduced thermogenesis, mouse metabolism profiling was performed. TrkA EC KO mice had lower cold tolerance, indicating brown adipocyte dysfunction (Figure 2D). As expected from this, indirect calorimetry data demonstrated reduced metabolic rate in TrkA EC KO mice (Figure 2E and Supplementary Fig. 2D). Finally, TrkA EC KO mice had lower glucose tolerance, also typical of brown adipocyte dysfunction (Supplementary Fig. 2E). Based on these observations, we conclude that endothelial NGF-TrkA signaling coordinates the function of nerves and vessels to support adipocyte thermogenesis and energy expenditure in BAT and SAT (Figure 2F).

### 3.3 Cell depletion via TrkA targeting in at compromises thermogenesis

To test if vascular TrkA can serve as a therapeutic target, we designed a compound targeting TrkA-expressing EC. We reasoned that depletion of the endothelium associated with thermogenic nerves may limit the ability of AT to maintain brown and beige adipocytes (Figure 3A). Based on the previously established approach [[30], [31], [32]], we synthesized a ‘hunter-killer’ peptide composed of a pro-apoptotic amphipathic peptide KLAKLAKKLAKLAK inactivating mitochondria [43] and the TrkA-binding PEP3 linked via aminohexanoic acid. As in our previous studies [31,48], the peptide was synthesized with all amino acids as d-enantiomers, which are not recognized by proteases and the immune system, to circumvent the problem of short half-life of peptides *in vivo* due to proteolytic degradation.

Based on regimens found to be safe and effective previously for other KLAKLAKKLAKLAK-based hunter killer peptides [[30], [31], [32]], we performed initial experiments with male C57BL/6 mice. Over two weeks, animals received seven metronomic subcutaneous injections of 1 mM D-BAT or of PBS (control). To assess if EC depletion leads to thermogenesis dysfunction, we subjected mice to cold tolerance test post-treatment. Mice treated with D-BAT displayed a decrease in cold tolerance, indicating jeopardized BAT and beige AT activity (Figure 3B). In contrast, mice treated with a control hunter-killer peptide D-WAT [31,32,48] did not display cold intolerance (data not shown). Organs were resected from mice 48 h after the last injection. The color of interscapular BAT depots was notably lighter, compared to control age-matched treated mice (Figure 3C). This indicated ablation of BAT vasculature and possible changes in adipocytes composing BAT. To confirm this, we performed IF analysis of tissue sections with antibodies against cleaved caspase 3, a marker of apoptosis. While no signal was observed in BAT of control (untreated) mice, apoptosis was abundant in BAT IB4+ cells of mice treated with D-BAT (Figure 3D). Cleaved caspase 3 signal was also observed in occasional IB4+ cells of SAT, but was at a background level in VAT, lung, and liver of D-BAT-treated mice (Figure 3D, Supplementary Fig. 3A). As expected, vascular density was significantly reduced by D-BAT treatment (Supplementary Fig. 3B). To confirm selectivity of D-BAT for EC, we used progeny of the cross between TIE2e-Cre and mTmG mice [39], in which membrane GFP (mG) becomes expressed in Cre-positive cells instead of membrane Tomato (mT) reporter. Primary plastic-adherent SVF from BAT of TIE2e-Cre; mTmG mice were treated with 100 nM D-BAT for 2 h in culture. Cleaved Caspase 3 IF was observed in mG + cells of TIE2e-Cre lineage (EC) but not in cells lacking mG expression (Supplementary Fig. 3C). *In situ*, cleaved caspase 3 was not detected in TH + nerves and was specific for the endothelium (Supplementary Fig. 3D). Apoptosis was also observed in BAT adipocytes, indicating that adipocyte survival depends on functional vasculature (Figure 3D). Expression of thermogenic protein UCP1 was not noticeably affected in remaining brown adipocytes (Figure 3E). However, co-staining for perilipin-1 revealed that brown adipocytes were partly replaced by adipocytes containing large lipid droplets in BAT of treated mice (Figure 3E). H/E staining of tissue sections from treated and control mice did not reveal differences in VAT or control organs, including liver and skeletal muscle (Supplementary Fig. 3E). However, there was a clear increase in brown adipocyte lipid droplet size induced by D-BAT (Figure 3F). Moreover, patches of beige adipocytes abundant in SAT of control mice were not observed upon D-BAT treatment (Figure 3F), which was confirmed by UCP1 IF (Supplementary Fig. 3F). Combined, these data indicate that thermogenic defect upon D-BAT treatment results from vasculature ablation and partial replacement of brown adipocytes with white in both BAT and beige AT.

### 3.4 TrkA-mediated cell depletion reduces metabolic rate and predisposes to obesity

Suppression of BAT and beige AT thermogenic activity has been reported to promote development of obesity and metabolic dysfunction [1]. Brown/beige adipocyte activity is known to be higher in females, which are also more resistant to DIO, relative to males [49]. After three weeks of treatment, D-BAT-injected and control C57BL/6 females did not have a notable difference in body weight (Supplementary Fig. 4A). EchoMRI analysis of body composition also did not reveal significant changes in lean and obese body mass (Supplementary Fig. 4B). Treated mice displayed a trend for increased food consumption, indicating that D-BAT is not toxic, although this was not statistically significant (Supplementary Fig. 4C). Circulating glucose levels were also unchanged in treated mice (Supplementary Fig. 4D). However, indirect calorimetry revealed that oxygen consumption was decreased upon treatment during both day and night (Supplementary Fig. 4E).

To further interrogate the consequences of D-BAT-induced metabolic suppression in females, we challenged them by HFD feeding for 3 months after treatment. Long term, food consumption was still not affected by D-BAT treatment (Figure 3G). However, mice treated with D-BAT and fed HFD began to display a decrease in cold tolerance, indicating that BAT activity was suppressed (Figure 3H). Consistent with that, indirect calorimetry revealed that mice treated with D-BAT had oxygen consumption decreased by two-fold during the day (Figure 3I). Respiratory exchange ratio (RER) calculation revealed an increase in treated animals, indicating that upon BAT inactivation mice metabolize less lipids as energy source (Figure 3I). After 3 months on HFD, mice treated with D-BAT had significantly higher body mass than control mice (Figure 3J). EchoMRI measurement of body composition revealed that fat body mass was increased in mice treated with D-BAT, while lean body mass was not significantly affected (Figure 3J). Inraperitoneal glucose injection revealed a decrease in glucose tolerance of D-BAT-treated mice, albeit very modest (Figure 3K). Consistent with that, insulin tolerance test (ITT) indicated that mice treated with D-BAT were more insulin-resistant than control mice (Figure 3L). Upon necropsy, an increase in lipid droplet size in BAT and a reduction in beige adipocyte content in periovarian AT and SAT was observed (Supplementary Fig. 4F). These data indicate that D-BAT treatment reduces metabolic activity, reduces glucose clearance, and promotes WAT expansion as a result of dysfunction in BAT and beige AT.

We previously reported increased PEP3 SAT homing in cold-conditioned mice [33]. We therefore predicted that upon SNS activation mice will be more responsive to D-BAT treatment. To mimic SNS activation, we treated female C57BL/6 mice with a β3-adrenergic agonist CL316,243 that induces browning in both SAT and VAT [24]. After that, mice received two subcutaneous injections of D-BAT, or PBS as control, over the next two days. Visual organ examination revealed that D-BAT treatment resulted in partial resorption of not only BAT and SAT, but also of perigonadal VAT in animals pre-treated with CL316,243, which was less pronounced in animals treated with D-BAT alone (Supplementary Fig. 5A). These observations were confirmed by quantification of weights for resected AT depots, which also revealed an additive effect of D-BAT and CL316,243 on AT depot size reduction (Supplementary Fig. 5B). Mice were then sacrificed and BAT, SAT and VAT were analyzed for apoptosis by cleaved caspase 3 IF. As shown in Supplementary Fig. 5C, apoptotic cell frequency in CL316,243-treated mice pre-treatment was increased in BAT and SAT, which was linked with notable vascular integrity disruption (Supplementary Fig. 5C). Apoptotic cells were absent in mice treated with D-BAT alone but detected in VAT of mice pre-treated with CL316,243 prior to D-BAT (Supplementary Fig. 5C). These data suggest that adrenergic SNS activation, promotes SAT and VAT sensitization to D-BAT by inducing endothelial TrkA expression. Confirming this, analysis of SVF from mice treated with CL316,243 demonstrated that in BAT the 70 kDa TrkA isoform was induced in a time-dependent manner (Supplementary Fig. 5D).

### 3.5 D-BAT treatment suppresses cancer-induced at browning and lipolysis

In mice, tumor growth induces lipolysis and browning in the surrounding AT, as shown by us [24] and others [17,28,50,51]. To test whether D-BAT can suppress these cancer-induced changes in peritumoral AT, we used an orthotopic mouse cancer model. Breast adenocarcinoma E0771 cells were grafted into the mammary fat pad of female C57BL/6 mice housed at ambient temperature. Six subcutaneous D-BAT or PBS injections were then performed during tumor growth. When the smallest tumors reached the diameter of 0.4 cm^3^, tissues were resected from all mice on the same day. Visual examination indicated a decrease in BAT (Figure 4A), which was confirmed by quantification of weights for resected AT depots (Figure 4B). We also observed a trend for tumor size reduction in response to D-BAT treatment, although it was not significant. Histopathological analysis by H/E staining demonstrated that, while tumor growth did not significantly induce browning in inguinal SAT distal to the tumor, patches of brown-like adipocytes were observed in belly AT adjacent to the tumors in PBS-injected (but not in D-BAT-treated) mice (Supplementary Fig. 6A). UCP1 IF, quantified in Supplementary Fig. 6B, confirmed these observations: areas of beige adipocytes strongly expressing UCP1 were abundant in AT proximal to tumors in control animals (Figure 4C–D). Importantly, this tumor-associated AT browning was not observed in mice treated with D-BAT (Figure 4C–D). We also quantified IF with antibodies against phosphorylated (serine 660) hormone-sensitive lipase (pHSL) to assess adipocyte lipolytic activity (Supplementary Fig. 6B). Analysis of interscapular BAT sections revealed high pHSL signal in control animals and its decrease in mice treated with D-BAT (Figure 4E). Expression of pHSL was also strong in clusters of beige adipocytes in tumor-proximal AT in PBS-injected animals, indicating that lipolysis is locally induced by tumor growth (Figure 4E). However, adipocytes in tumor-proximal AT were larger and did not express pHSL in mice treated with D-BAT (Figure 4E). Compared to controls, more cancer cells of D-BAT-treated mice had low expression of hydroxyacyl-CoA dehydrogenase (HADHA), the enzyme catalyzing the last three steps of mitochondrial fatty acid (FA) beta-oxidation (Supplementary Fig. 6C). This could result from adipocyte lipolysis and FA mobilization inhibition. Because FA are utilized by tumors as energy substrate and membrane building blocks [52] a decrease of FA availability for cancer cells could explain reduced tumor growth. These results indicate that D-BAT treatment suppresses tumor growth-induced AT lipolysis and FA mobilization.

## 4 Discussion

Here, we used PEP3, a peptide homing to BAT and beige AT [33], to identify an isoform of TrkA as the endothelial PEP3 receptor. We show that PEP3 binds to TrkA by mimicking a segment of NGF. PEP3 is likely to be a weak ligand, but, nonetheless, can inhibit the activating action of NGF *in vitro*. We provide evidence that endothelial TrkA mediates neuro-vascular remodeling along with underlying thermogenic adipocyte differentiation. Functional connections between sympathetic ganglia and BAT are established during development and maintained in adulthood through the release of NGF and other neurotrophins by brown preadipocytes [53]. The NGF-TrkA co-receptor, p75, is also produced by brown adipocytes [54]. NGF increases BAT activity [55], whereas administration of neutralizing NGF antiserum reduces it [56]. It has been reported that NGF-TrkA signaling guides SNS axons and supports BAT innervation [57]. However, the possibility that NGF expressed by brown/beige adipocytes may be important for vascular function has not been previously explored. Here, by analyzing vascularization and endothelial sprouting, we show that TrkA-negative EC do not have an intrinsic defect in angiogenic function. However, the alignment of blood vessels and nerves is disrupted in AT of TrkA EC KO mice. We propose that NGF-TrkA signaling in BAT/beige endothelium and SNS couples angiogenesis with innervation conducive of catecholamine secretion that facilitates AT beiging (Figure 2F). However, we cannot exclude the direct (nerve-independent) contribution of the defect in endothelial cells to the phenotype.

Although expression of TrkA in EC has been reported [58], our discovery of differential expression of TrkA specifically in BAT and beige AT vascular beds is novel. Data throughout the manuscript indicate that endothelial TrkA is indeed expressed in SAT of mice housed at RT, which are not thermoneutral and have beige AT. While full-length glycosylated TrkA has a MW of over 110 kDa, its truncated isoforms generated by ectodomain cleavage have been previously detected by Western blotting [59]. The molecular mechanism generating the endothelium-specific 70 kDa TrkA isoform in AT remains to be determined. It is also not clear how NGF-TrkA signaling is integrated with the known molecular effectors of angiogenesis. Previously, VEGF has been found to mediate vascularization during the conversion of AT from white to beige [29]. Interestingly, NGF-TrkA signaling has been reported to increase VEGF expression via induction of HIF-1α [60]. The importance of HIF-1α/VEGF axis in AT and its link with SNS activation is well established [29,47,61,62]. Our findings further position TrkA as a player in the signaling cascade linking SNS and vascularization. Despite the therapeutic potential of NGF as a treatment for peripheral neurodegenerative disorders, clinical trials have been disappointing partly due to its quick clearance and systemic side effects, such as hyperalgesia [63]. We propose that homing of PEP3 to BAT and SAT could be harnessed to utilize TrkA as an endothelial receptor for targeting anti-diabetic therapy. Pharmacological TrkA activation restricted to AT could be developed as a new approach to metabolism improvement through inducing beige adipocyte differentiation in SAT.

Our group has discovered a number of tissue-homing peptides binding to differentially expressed vascular molecules [30,41,[64], [65], [66], [67]]. These peptides have been useful as delivery vehicles in therapy targeting applications [30,31,43,68]. Here, we used PEP3 as a vehicle to target brown and beige AT *via* vascular TrkA in an experimental therapy setting. We show that a PEP3-derived hunter-killer peptide D-BAT induces cell death in BAT and beige AT endothelium, which results in brown adipocyte cell death and AT remodeling. As a result of thermogenic AT function disruption by D-BAT, adipocyte whitening predisposes to obesity (Figure 3A). Our data are consistent with the phenotype observed upon brown adipocyte ablation *via* a transgenic toxigene approach [13]. Our hunter killer-peptide design had been previously used for other receptor-binding peptides and taken to the clinic [64,69]. A peptide targeting WAT has been shown to reverse obesity and increase metabolism in several animal models [30,68]. Another peptide targeting white adipocyte progenitors prevents obesity development and also increases metabolic rate [31]. The opposite metabolic effect of D-BAT, not previously observed with other hunter-killer peptides, indicates its efficacy and specificity.

Positive metabolic effects resulting from AT browning in animal models have given hopes that activation of AT browning could be developed as a diabetes treatment [5,15]. However, excessive AT lipolysis and browning have also been linked with adverse hypermetabolic conditions [20] and lipodystrophy [70]. While it is not clear if cachexia in human cancer patients involves UCP1-mediated uncoupled thermogenesis activation, AT lipolysis in cancer is clearly linked with tumor aggressiveness [16,18,19]. Here, we provide evidence that targeting AT lipolysis in tumor proximity can affect tumor metabolism. Our study is a step toward developing new approaches to control AT function.

## Author contributions

M.G.K. conceived/designed the experiments and wrote the manuscript. A.C.D., Z.G. and Y.Y. designed and performed the experiments, analyzed data, and edited the manuscript.
