# Supplementary material for: Endothelial TrkA coordinates vascularization and innervation in thermogenic adipose tissue and can be targeted to control metabolism
Source: Mol Metab. 2022 Jul 11;63:101544. doi: 10.1016/j.molmet.2022.101544 (PMC9310128; doi:10.1016/j.molmet.2022.101544)
Supplement: Multimedia component 1 [file mmc1.pdf]

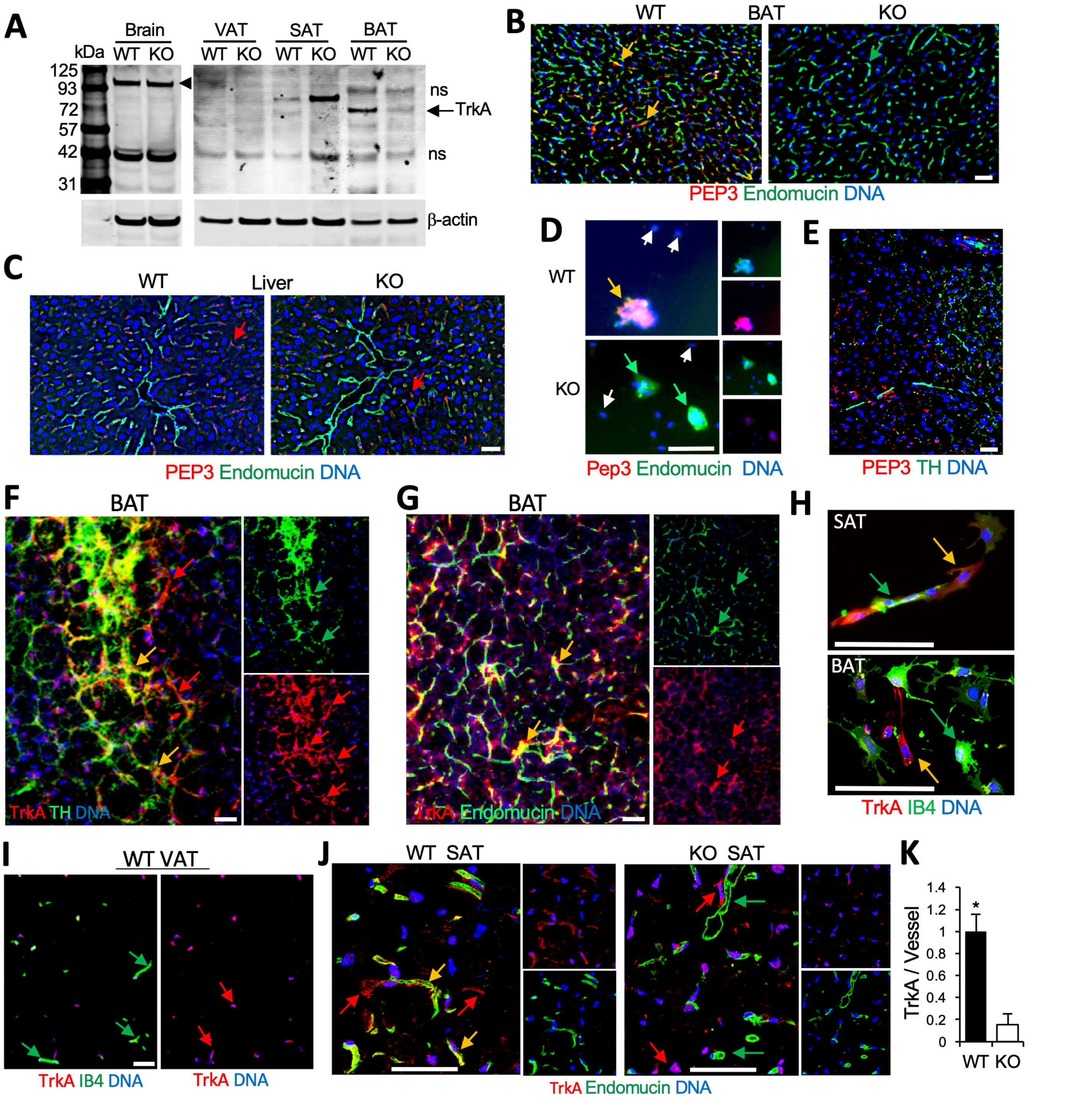

Supplementary Figure 1: Endothelial TrkA is the target of PEP3 in AT. (**A**) Immunoblot showing reduced presence of truncated TrkA (arrow) in BAT and SAT of TrkA EC-KO (KO) mice and equal presence of full-length TrkA (arrowhead) in brain of WT and KO mice. β-actin: loading control; ns: nonspecific bands. (**B**) BAT sections of mice injected with Phage-PEP3 subjected to anti-phage/anti-endomucin IF. Note vascular homing of phage in WT but not in EC KO mice. (**C**) Liver sections of mice injected with Phage-PEP3 subjected to anti-phage/anti-endomucin IF. Note comparable phage liver trapping in both WT and KO mice. (**D**) Mice were injected with 0.5 mg of biotinylated PEP3. After 1 hr circulation, BAT SVF was isolated and adherent cells were fixed and subjected to Strepatavidin-Cy3 (red) and anti-endomucin (green) IF. Yellow: PEP3 signal in endothelial colonies from WT mice but not KO littermates. White arrows: endomucin-negative stromal cells lacking PEP3. (**E**) SAT sections of WT mice injected with Phage-PEP3 subjected to anti-phage/anti-TH IF showing a lack of co-localization. (**F**) Whole mounts of WT BAT subjected to IF demonstrating TrkA co-localized (yellow) and not co-localized (red) with nerves (TH+, green). (**G**) Whole mounts of WT BAT subjected to IF demonstrating TrkA co-localized (yellow) and not co-localized (red) with blood vessels (endomucin+, green). (**H**) IF on SVF from WT mice of demonstrating TrkA expression in some but not all EC co-stained with IB4. (**I**) IF of VAT from WT mice demonstrating a lack of TrkA co-localization with EC (IB4+). (**J**) Confocal IF of demonstrating TrkA co-localization with EC (IB4+) in WT but not KO mice. (**K**) Quantification (mean ± SEM; **P*<0.01) of TrkA signal association with EC in WT and KO mice. Scale bar: 50 μm.

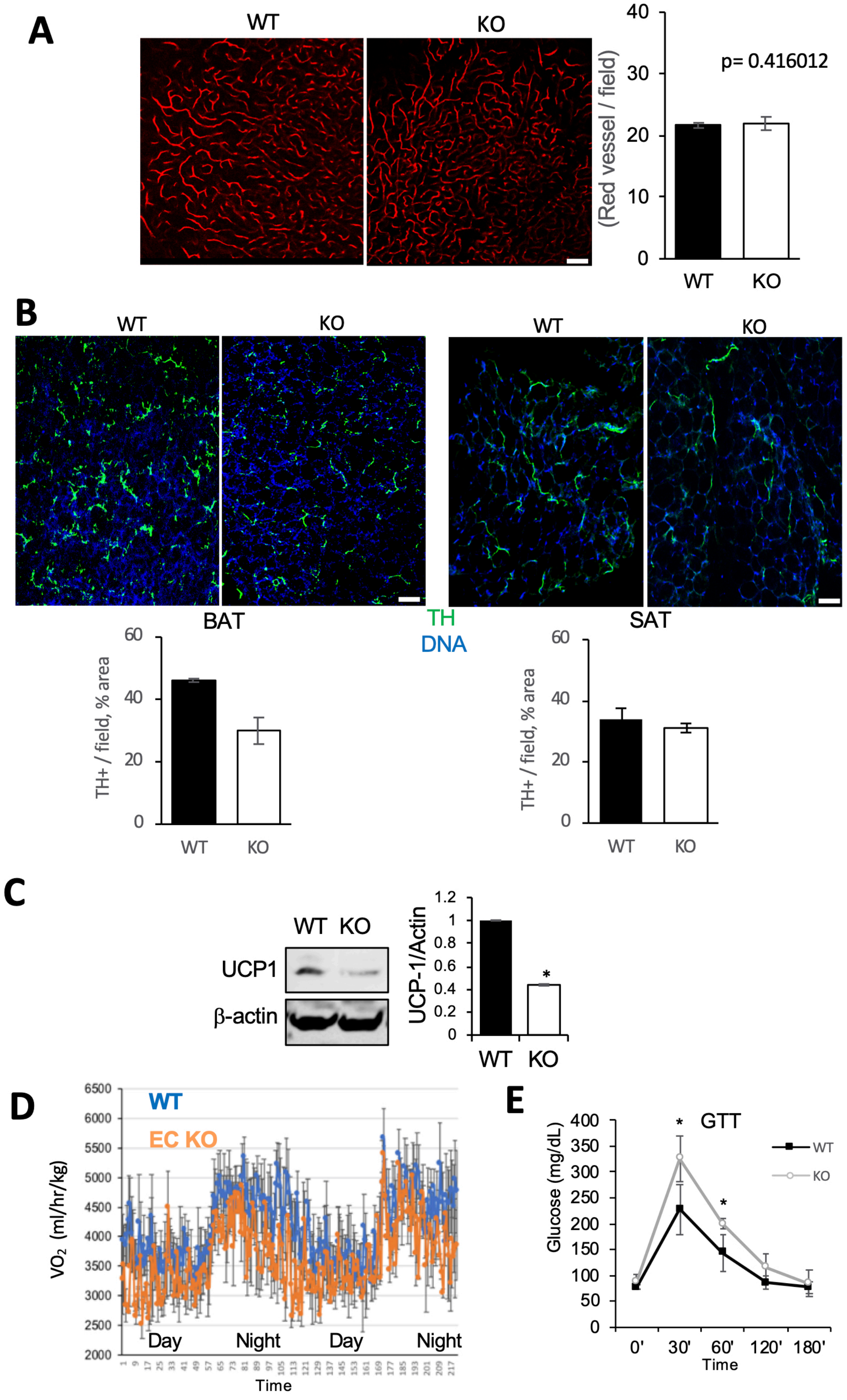

Supplementary Figure 2: Endothelial TrkA function in AT assessed in TrkA EC-KO (KO) mice. (**A**) Vascular perfusion in WT and KO mice analyzed upon iv infusion of Red FluoroSphere microbeads in SAT whole mounts. Quantification is on the right. (**B**) BAT and SAT of WT and KO littermates subjected to TH IF revealing comparable nerve density in AT of WT and KO mice and decreased nerve branching in BAT of KO mice. Quantification is below. (**C**) Western blot showing lower (mean ± SEM; **P*<0.05) UCP1 expression in SAT of KO mice. β-actin: loading control. Graph: data quantification. (**D**) Metabolic chamber data showing reduced oxygen consumption by KO mice. (**E**) Lower glucose tolerance (mean ± SEM; **P*<0.05) of male KO mice measured after i.p. glucose injection into overnight-fasted mice over 180 min. Scale bar: 50 μm.

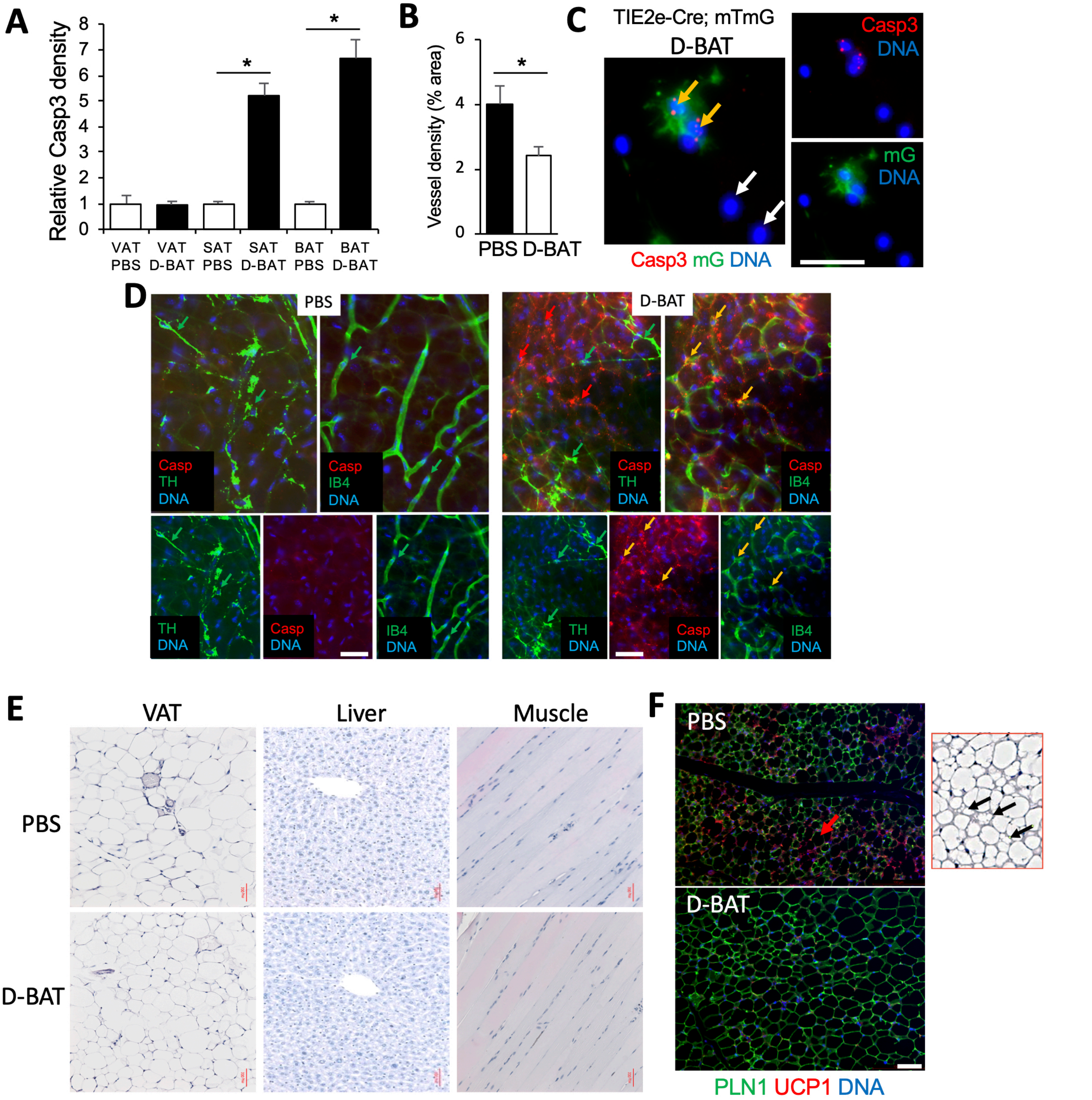

Supplementary Figure 3: D-BAT induces apoptosis selectively in EC of thermogenic AT. (**A**) Quantification (mean ± SEM; *P<0.01) of caspase 3 IF in data used for Figure 3D demonstrating apoptosis induction in BAT and SAT. (**B**) Quantification (mean ± SEM; *P<0.01) of IB4+ vasculature in BAT of mice treated with D-BAT, compared to PBS control. (**C**) SVF isolated from BAT of TIE2e-Cre; mTmG mice were treated with 100 nM of D-BAT for 2 hours. Cleaved Caspase 3 IF is observed in EC of TIE2e-Cre^{+} lineage (yellow arrows) but not in cells lacking mG expression (white arrows). Cleaved Caspase-3 antibody: CellSignaling cat. # 9661, 1:75. Secondary: Jackson ImmunoResearch Cy5-conjugated donkey anti-goat IgG, cat. # 705-175-147, 1:200. In RGB images, Cy5 fluorescence is pseudo-colored red to replace mT fluorescence (not shown). (**D**) Mice were injected with PBS or D-BAT; after 12 hr BAT whole mount was subjected to Cy3 IF for cleaved Caspase 3 (Casp), Alexa488 IF for TH, and IB4-biotin / Cy5-strepatavidin. In 4-color fluorescence, Cy5 fluorescence was pseudo-colored green for co-localization with Casp. Note the lack of Casp signal co-localization with TH and co-localization with IB4 in mice treated with D-BAT. (**E**) C57BL/6 mice housed at RT and treated chow were treated with D-BAT. Shown are representative images of H/E-stained paraffin sections of VAT, liver and gastrocnemius skeletal muscle, which are all not affected by D-BAT. (**F**) SAT of mice subjected to PLN1 and UCP1 IF and showing reduced presence of beige (UCP1+) adipocytes (arrow) in mice treated with D-BAT. Inset: high magnification H/E from Fig. 3F. Scale bar: 50 µm.

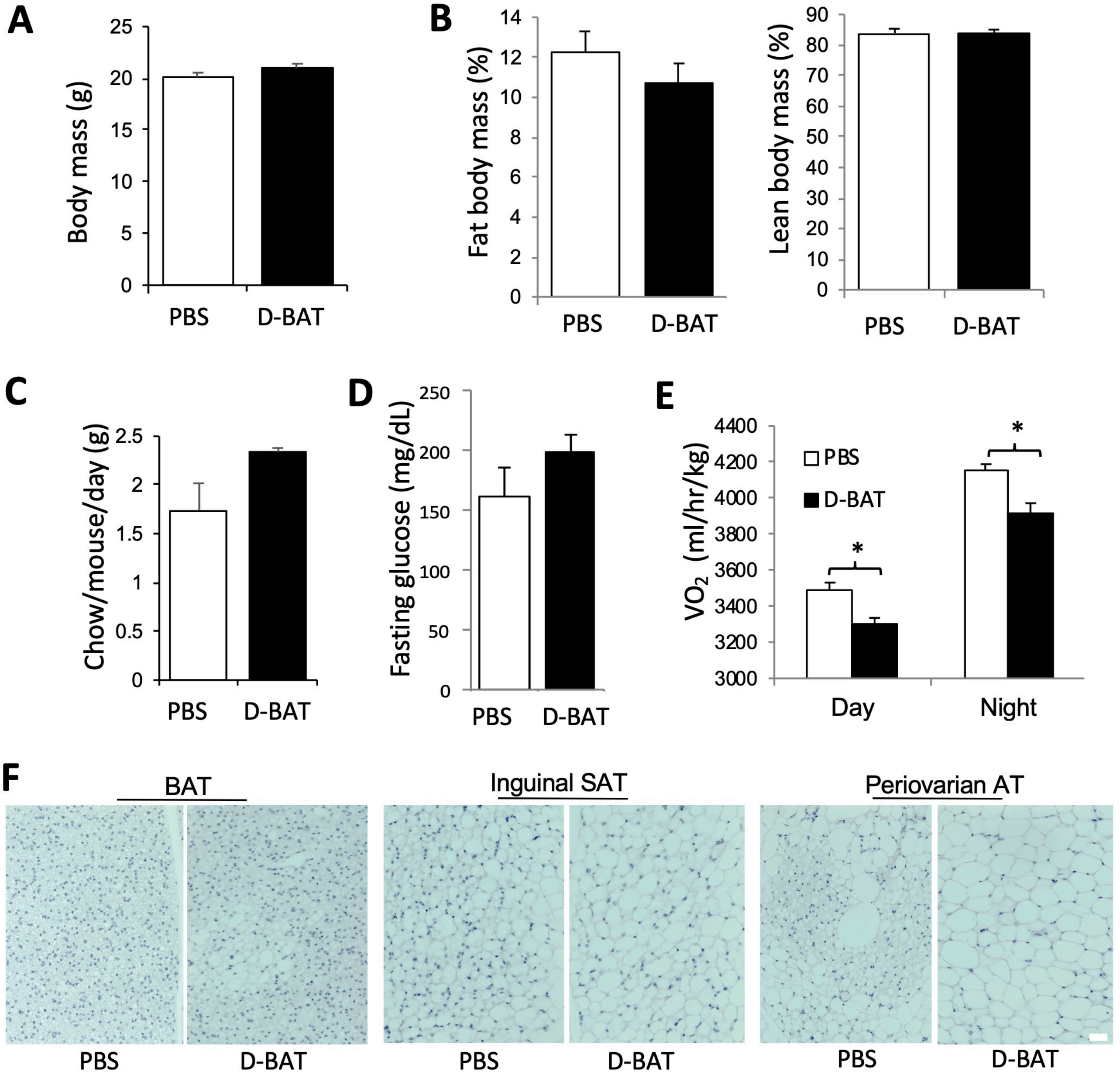

Supplementary Figure 4: D-BAT treatment effect on energy expenditure in chow-fed female mice. C57BL/6 female mice were analyzed after 10 sc D-BAT injections over three weeks. (**A**) Body mass. (**B**) Body composition measured by EchoMRI. (**C**) Food consumption. (**D**) Fasting glucose. (**E**) Oxygen consumption during day and night decreased by upon D-BAT treatment. For all graphs, plotted are mean ± SEM; **P*<0.05, (Student's t-test). (**F**) Representative images of H/E-stained paraffin sections of BAT, SAT and periovarian AT from representative mice treated revealing that D-BAT decreases beige adipocyte content. Scale bar: 50 µm.

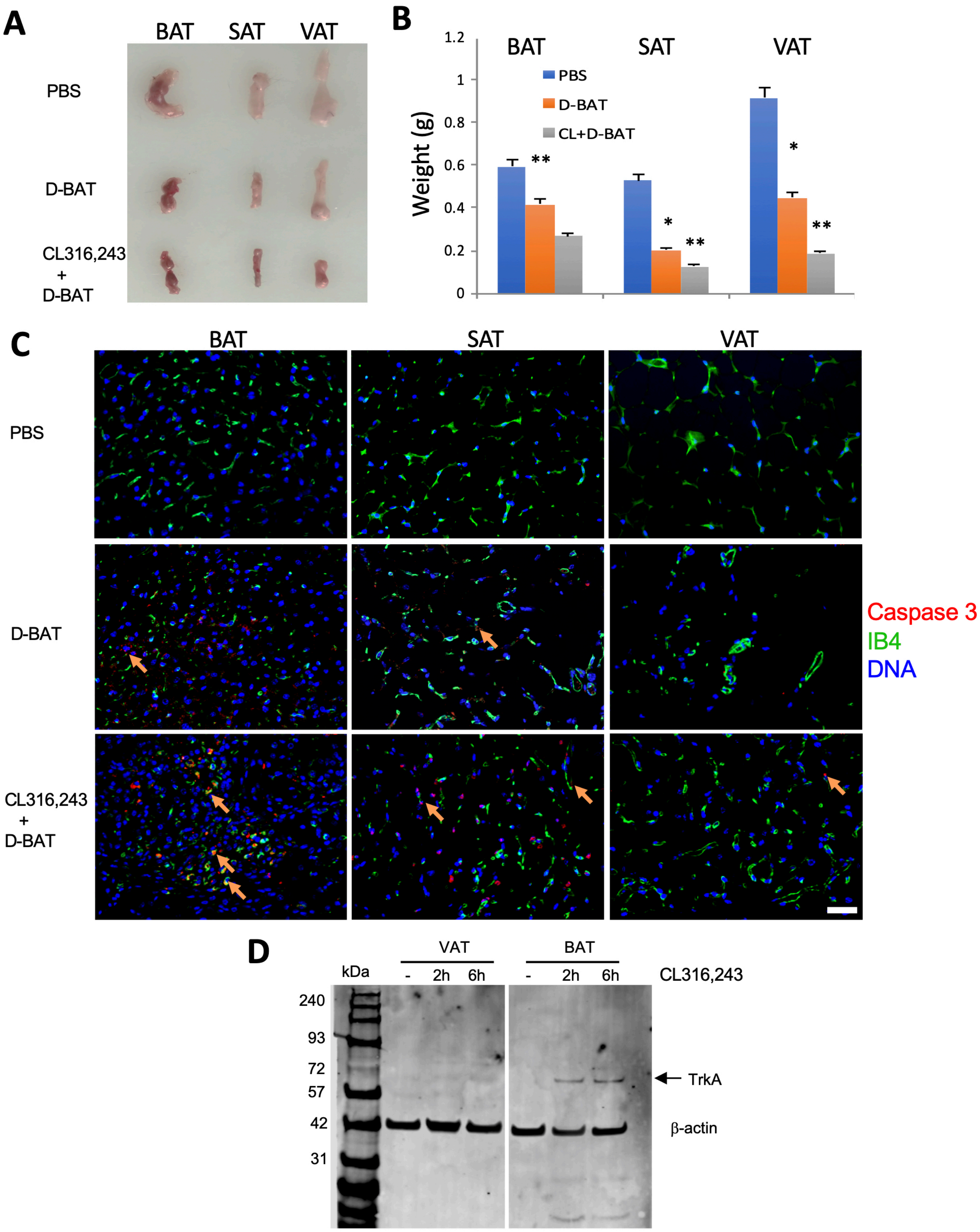

Supplementary Figure 5: Effect of D-BAT on AT is enhanced by SNS activation. C57BL/6 female mice (2 months old) were injected i.p. daily for five days with 1 mg/kg CL316,243 (CL) to activate AT beiging, after which two 0.1 ml D-BAT (1 mM) or PBS (control) injections were performed over a period of two days. Tissues were recovered a day after the last injection. (**A**) Interscapular BAT, inguinal SAT and gonadal VAT resected from mice. (**B**) Quantification of organ weights in (A). Plotted are mean ± SEM; **P*<0.05, ***P*<0.01 (Student's t-test). (**C**) IF analysis of paraffin tissue sections from D-BAT-treated and control mice for caspase 3 cleavage (apoptosis marker) upon co-staining with isolectin B4 (green) marking endothelium. Note increased AT apoptosis frequency in CL / D-BAT-treated mice. Scale bar: 50 µm; nuclei are blue. (**D**) Western blot showing increased presence of the low molecular weight TrkA isoform (arrow) in BAT of WT mice treated with CL316,243. β-actin: loading control.

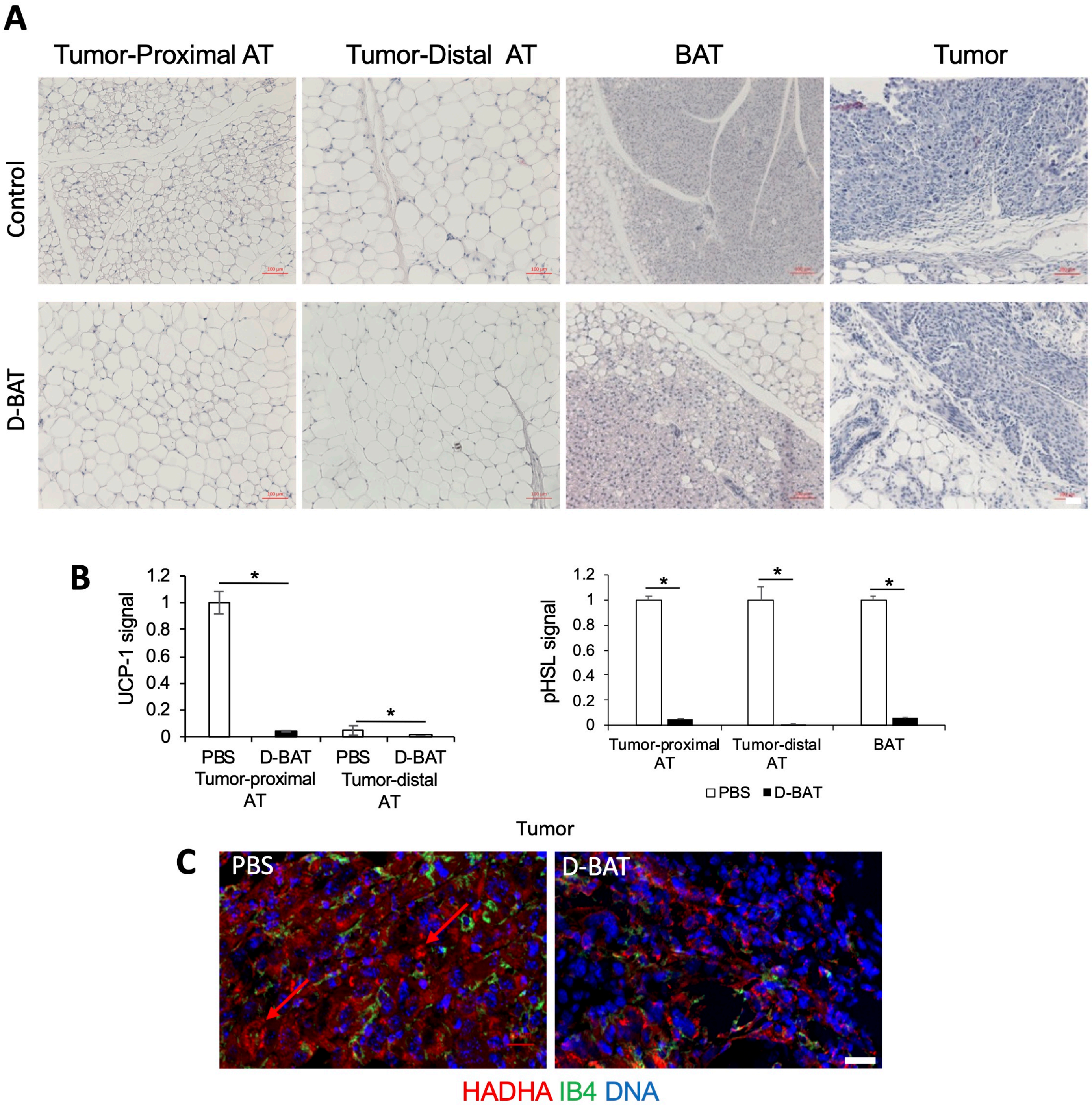

Supplementary Figure S6: D-BAT attenuates tumor-associated AT browning and lipolysis. C57BL/6 female mice received ten 0.1 ml injections of D-BAT (0.5 mM) over three weeks. Then, mice were grafted with tumors into mammary fat pad and received six more D-BAT injections over two subsequent weeks prior to tissue resection. (**A**) Representative images of H/E-stained paraffin sections of AT, BAT, and tumors from representative mice. (**B**) Quantification of data from Figures 4D and 4E assessing UCP1 and pHSL expression in distinct AT depots upon D-BAT treatment of tumor-bearing mice. Plotted are mean ± SEM; **P*<0.01, (Student's t-test). (**C**) IF analysis showing lower FAO in cancer cells (HADHA expression) and vascularization (EC staining with IB4) in tumors of control and D-BAT-treated mice. Scale bar: 50 μm.
